# The ARF Tumor Suppressor Regulates Bone Remodeling and Osteosarcoma Development in Mice

**DOI:** 10.1371/journal.pone.0015755

**Published:** 2010-12-30

**Authors:** Daniel A. Rauch, Michelle A. Hurchla, John C. Harding, Hongju Deng, Lauren K. Shea, Mark C. Eagleton, Stefan Niewiesk, Michael D. Lairmore, David Piwnica-Worms, Thomas J. Rosol, Jason D. Weber, Lee Ratner, Katherine N. Weilbaecher

**Affiliations:** 1 Division of Molecular Oncology, Department of Medicine, Washington University School of Medicine, St. Louis, Missouri, United States of America; 2 College of Veterinary Medicine, The Ohio State University, Columbus, Ohio, United States of America; 3 Center for Retrovirus Research, Department of Veterinary Biosciences, The Ohio State University, Columbus, Ohio, United States of America; 4 Molecular Imaging Center, Mallinckrodt Institute of Radiology, Department of Developmental Biology, Washington University School of Medicine, St. Louis, Missouri, United States of America; UCLA and Cedars-Sinai Medical Center, United States of America

## Abstract

The ARF tumor suppressor regulates p53 as well as basic developmental processes independent of p53, including osteoclast activation, by controlling ribosomal biogenesis. Here we provide evidence that ARF is a master regulator of bone remodeling and osteosarcoma (OS) development in mice. *Arf^-/-^* mice displayed increased osteoblast (OB) and osteoclast (OC) activity with a significant net increase in trabecular bone volume. The long bones of *Arf^-/-^* mice had increased expression of OB genes while *Arf^-/-^* OB showed enhanced differentiation *in vitro*. Mice transgenic for the Tax oncogene develop lymphocytic tumors with associated osteolytic lesions, while Tax+*Arf^-/-^* mice uniformly developed spontaneous OS by 7 months of age. Tax+*Arf^-/-^* tumors were well differentiated OS characterized by an abundance of new bone with OC recruitment, expressed OB markers and displayed intact levels of p53 mRNA and reduced Rb transcript levels. Cell lines established from OS recapitulated characteristics of the primary tumor, including the expression of mature OB markers and ability to form mineralized tumors when transplanted. Loss of heterozygosity in OS tumors arising in Tax+*Arf^+/-^* mice emphasized the necessity of ARF-loss in OS development. Hypothesizing that inhibition of ARF-regulated bone remodeling would repress development of OS, we demonstrated that treatment of Tax+*Arf^-/-^* mice with zoledronic acid, a bisphosphonate inhibitor of OC activity and repressor of bone turnover, prevented or delayed the onset of OS. These data describe a novel role for ARF as a regulator of bone remodeling through effects on both OB and OC. Finally, these data underscore the potential of targeting bone remodeling as adjuvant therapy or in patients with genetic predispositions to prevent the development of OS.

## Introduction

ARF (p14^ARF^ in humans, p19^ARF^ in mice) is a tumor suppressor and a key sensor of hyperproliferative signals such as those from the Ras and Myc oncoproteins [1]. In response to oncogenic stress, ARF causes cell-cycle arrest in G1 and G2/M and is associated with increased p53, p21cip and MDM2 expression [1], [2]. ARF mediates cell-cycle arrest by directly binding to MDM2 and sequesters it in the nucleolus. Sequestration of MDM2 stabilizes and activates p53 which then blocks cellular proliferation [2], [3]. Alternatively, ARF inhibits cellular growth in a p53-independent manner by regulating ribosomal biogenesis through nucleophosmin [3].

*Arf*-deficient mice develop undifferentiated sarcomas (rarely osteosarcomas), lymphomas, and carcinomas with a mean life expectancy of ∼9 months [4]. Mice lacking both *Arf* and *p53* exhibit a tumor spectrum distinct from that observed in single knockout animals, implying that ARF and p53 can act independently to suppress tumor formation [3]. Not surprisingly, expression of oncogenes in *Arf*-/- transgenic mice affects the timing and characteristics of the tumors that arise. For example, *Arf^-/-^* mice transgenic for Eµ-*myc* developed aggressive B cell lymphomas with a dramatic acceleration in onset and increased mortality when compared to *Arf^+/+^* Eµ-*myc* mice [5]. Additionally, c-myc overexpression in *Arf^-/-^* bone marrow resulted in the rapid development of acute myeloid leukemia [6].

ARF possesses activities that are independent of its role as a tumor suppressor. *Arf*-deficient mice succumb to blindness soon after birth. In this case, the lack of programmed cell death within the hyaloid vasculature leads to an accumulation of perivascular cells and destruction of the retina and lens [7]. We have previously described that *Arf* deficiency leads to increased osteoclast (OC) function and accelerated differentiation through nucleophosmin, independent of p53 [8]. We now report that *Arf*-deficient mice also develop enhanced osteoblast (OB) function and that the co-regulation of OB and OC in the absence of ARF leads to constitutively increased bone remodeling in vivo.

The role of ARF in bone remodeling carries implications for cancer biology. p53, which is regulated by ARF, has also been demonstrated to regulate OB function [9]. Moreover, the bone microenvironment is known to support tumor growth in leukemia, myeloma and solid tumor bone metastasis [10], [11], [12], [13]. To pursue the implications of accelerated bone remodeling on tumor development we bred *Arf* ^-/-^ mice with mice expressing the viral oncogene Tax [14]. Since the lymphoid tumors that arise in Tax mice cause significant bone loss (osteolysis) [15], [16], we hypothesized that crossing *Arf^-/-^* mice (prone to accelerated bone remodeling) with Tax mice (prone to osteolytic malignancies) would exacerbate tumorigenesis and tumor mediated osteolysis. Unexpectedly, the Tax+*Arf* ^-/-^ mice became highly susceptible to development of spontaneous osteosarcoma. Osteosarcoma (OS) is the most common primary bone malignancy, with a peak incidence in adolescence, a time of high bone remodeling, and 5-year survival rates in patients with metastatic or relapsed disease of <20% [17].

p53 mutations and Rb loss is common in human OS [17] and recent mouse models have provided clear evidence that OB-restricted deletion of p53 and Rb leads to OS [18], [19]. ARF loss is also common in human OS [17], [20]. We found that the OS that spontaneously arose in Tax+*Arf^-/-^* mice recapitulates the malignancy described in the p53/Rb OB-specific deletion models.

We further hypothesized that the increased bone remodeling in *Arf^-/-^* mice contributed to OS development and provide evidence that zoledronic acid, a bisphosphonate inhibitor of bone resorption, prevented or delayed onset of OS in Tax+*Arf^-/-^* mice. These data demonstrate that ARF regulates bone remodeling through cell autonomous effects on both OB and OC and introduce Tax+*Arf^-/-^* mice as a new model of spontaneous OS. This discovery implicates bisphosphonates as novel therapeutics for targeted prevention of OS after resection of primary OS tumors or in patients with genetic cancer predisposition syndromes.

## Methods

### Animals

*Arf^-/-^* mice on a C57BL/6 background [21] were intercrossed with transgenic mice expressing HTLV-1 Tax under the human granzyme B promoter on a C57BL/6 x FVB background (Tax+; previously described [14]). In all experiments, littermate *Arf^+/+^*, *Arf^-/-^*, Tax+ and Tax+*Arf^-/-^* mice on a C57BL/6 x FVB mixed background were used. In some experiments, *Arf^-/-^* mice were crossed with TAX-LUC mice, a strain with granzyme B dependent Tax expression driving firefly luciferase via the Human T-cell lymphotropic virus type I long terminal repeat promoter, thereby providing a bioluminescent readout of Tax-dependent early tumor development [22]. NOD-SCID-IL2γR^-/-^ (The Jackson Laboratory, Bayr Harbor, Maine) mice were used for experiments involving tumor allografts. The use of murine models and tissues in this study was carried out in strict accordance with the recommendations in the Guide for the Care and Use of Laboratory Animals of the National Institutes of Health. Mice were housed under pathogen-free conditions according to the guidelines of the Division of Comparative Medicine and all experiments were approved by the Animal Studies Committee, Washington University School of Medicine under protocols 20100151 (K.N.W.) and 20100026 (L.R.)

### Micro–computed tomography

Femurs were suspended in agarose and femoral metaphyses were scanned by micro–computed tomography (µCT; µCT-40; Scanco Medical) as described previously [23], [24]. For image acquisition, the femurs were placed in a 17-mm holder and scanned. The trabecular region was selected using contours inside the cortical shell on each image. The growth plate was used as a marker to determine a consistent location to start analysis and 40 slices were analyzed. A three-dimensional cubical voxel model of bone was built, and the calculations were made as follows: apparent bone mineral density (calibrated against a hydroxyapatite phantom), relative bone volume over total bone volume (BV/TV), trabecular number and thickness.

#### Bioluminescence imaging

Early tumor development and myloperoxidase (MPO) activity were monitored by non-invasive bioluminescence imaging of anesthetized mice (isoflurane inhalation) following the administration of D-luciferin or luminol as described ([22], [33] respectively), in an IVIS 100 instrument (Caliper Life Sciences, Alameda, CA).

### *In vivo* calcein labeling and calculation of bone formation rate

Mice were intraperitoneally injected with 20 mg/kg of calcein (Sigma-Aldrich, St. Louis, MO) in a 2% sodium bicarbonate solution. Mice were labeled 7 days and 2 days prior to sacrifice. Calveria were fixed in 70% ethanol and embedded in methylmethacrylate and sectioned. This labeling allowed for evaluation of bone surface (BS), single labeled surface (sLS) and double labeled surface (dLS). These measurements were then used to calculate bone formation rate (BFR/BS) per year as previously described [25]. Both the concave and convex surfaces of each bone were evaluated separately using a fluorescence microscope (Olympus BX51, Center Valley, PA). Analysis of bone formation rate (BFR) per bone surface (BS) was performed used Bioquant Osteo (Bioquant Image Analysis Corp.).

#### Serum osteocalcin

Serum was isolated from animals and a sandwich ELISA based assay for murine osteocalcin was performed according to manufacturer's instructions (Biomedical Technologies Inc, Stoughton, MA).

### RT-PCR and quantitative RT-PCR

RNA was isolated from in vitro differentiated OB or TAN OS tumor cells using the Qiagen RNeasy mini kit (Qiagen, Valencia, CA). To isolate RNA from whole bones or whole OS, bone marrow was removed and bones were mechanically crushed with a mortar and pestle and RNA extracted using TRIzol reagent (Invitrogen, Carlsbad, CA) according to standard protocol. 2 µg of RNA were subjected to DNase I digestion (Invitrogen) and RT-PCR was carried out using SuperScript III First-Strand Synthesis system (Invitrogen) according to manufactures instructions. 100 ng of cDNA template were used for each reaction. Qualitative RT-PCR was performed using RedTaq (Sigma-Aldrich, St. Louis, MO) and quantitative RT-PCR was performed using iQ SYBR Green Supermix (Bio-Rad, Hercules, CA) on a CFX96 Real-Time PCR detection system (Bio-Rad). A list of primers utilized can be found in Table S1.

#### *In vitro* differentiation of osteoblasts

Whole bone marrow was flushed from the femurs and tibias. The resulting BM of one mouse was plated onto one 150 mm tissue-culture coated dish in αMEM media (GIBCO) containing 20% FBS, L-Glutamine, and 1% Pen-Strep (plating media). Non-adherent cells were removed after 2 days. Adherent bone marrow stromal cells were harvested with trypsin/EDTA at 70% confluence. For differentiation assays, 1×10^5^ cells/well were plated into a 24 well plate in plating media. For RNA isolation, 4×10^5^ cells/well were plated into a 6 well plate. The following day or when cells reached confluence, the plating media was replaced with osteogenic media (αMEM media, 20% FBS, L-Glutamine, 1% Pen-Strep, 50 µg/ml ascorbic acid, and 10 mM β-glycerophosphate). Cells were supplied fresh media every 7 days for the duration of the assay. At indicated timepoints, cell monolayers were washed with PBS and fixed in 10% neutral buffered formalin. Alkaline Phosphatase staining was carried out with Fast Blue RR salt (Sigma-Aldrich). 0.4% Alizarin Red solution or Von Kossa staining (0.25 g AgNO_3_ in 5 ml water) was used to detect mineralized matrix.

#### Radiography

Development of OS was monitored by serial x-ray imaging (Faxitron, Buffalo Grove, IL). Prior to imaging, mice were anesthetized with a cocktail of Ketamine/Xylazine.

#### Histology

Bones or OS were fixed in 4% paraformaldehyde and decalcified for 14–21 days in 14% EDTA. Paraffin-embedded sections were stained with H&E, TRAP, or immunohistochemical stains for Tax [22] and nucleophosmin [26] as previously described.

### Establishment of Tax+*Arf^-/-^* osteosarcoma cell line (TAN)

The TAN (Tax^+^ ARF null) tumor cell line was isolated from a spontaneous mandibular OS that arose in a Tax+*Arf^-/-^* mouse. All soft tissue was removed and the calcified tumor was mechanically digested into small pieces using bone rongeurs and a razor blade under sterile conditions. The small bone fragments were plated onto tissue-culture coated dishes in 10% RPMI-1640 (Sigma) media supplemented with 10% fetal calf serum and 1% Pen-Strep. After 1 week, non-adherent cells and bone fragments were removed. The adherent tumor cells were propagated.

#### TAN cell transplants

Mice were anesthetized and 1×10^4^ TAN cells in 50 µL PBS were injected into the left tibia. PBS was injected into the right tibia as an internal control. Animals were radiographed in two dimensions using an X-ray system to confirm intratibial placement of the needle (Faxitron Corp). Alternatively, 1×10^6^ TAN cells in 100 µL PBS were injected in the tail vein and monitored by radiography.

#### Zoledronic acid (ZA) dosing

ZA (Novartis Pharma AG, Basel, Switzerland) was administered subcutaneously at a dose of 0.75 µg/mouse/week (approx. 30 µg/kg) [27] or phosphate-buffered saline (PBS) starting at 1 month of age to Tax+*Arf^-/-^* mice. ZA dosing was continued on a weekly basis until 9 months of age or until the animal was moribund and sacrificed. This dosing schedule of ZA was designed to produce drug levels similar to those achieved with the clinical dosing regimen of 4 mg Zometa® for the treatment of bone metastases. OS development was monitored by manual palpation and confirmed by radiography.

#### Statistical Analysis

All experiments were analyzed using Student's *t*-test or one-way ANOVA in the case of experiments with greater than three experimental groups. In calculating two-tailed significance levels for equality of means, equal variances were assumed for the two populations. Kaplan-Meier plots were analyzed by the Log-rank (Mantel-Cox) Test. Results were considered to reach significance at p<0.05 and are indicated with an asterisk (*).

## Results

### Loss of the *Arf* tumor suppressor resulted in increased bone formation and enhanced osteoblast differentiation

**Figure 1:**
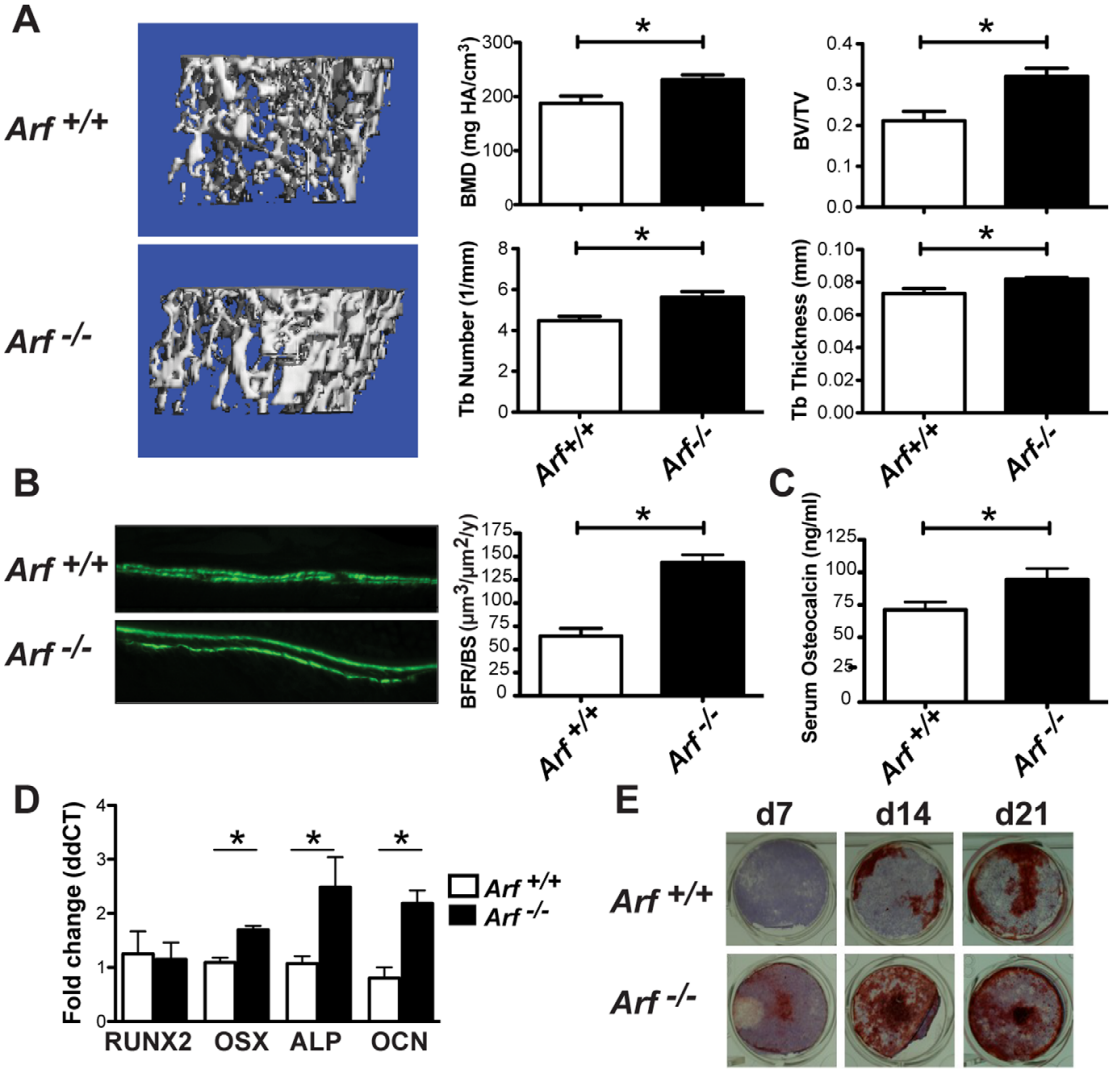
Loss of the *Arf* tumor suppressor resulted in increased bone formation and enhanced osteoblast differentiation. a) µCT analysis of tibias from 8-week-old female *Arf* ^+/+^ versus *Arf^-/-^* littermates (n = 6/genotype) mice. Bone mineral density (p = 0.0289), trabecular bone volume (BV/TV) (p = 0.0074), trabecular number (p = 0.0117) and trabecular thickness (p = 0.0252) were significantly increased in *Arf^-/-^* tibias. b) Bone formation was visualized by double calcein labeling 7d and 2d prior to sacrifice and visualized in the calveria. The distance between labels is directly proportional to bone formation. *Arf^-/-^* mice showed a significant increase in bone formation rate per bone surface (p = 0.0211). c) Serum osteocalcin levels were significantly increased in *Arf^-/-^* mice (p = 0.0376). d) Long bones of 8-week-old *Arf^-/-^* mice expressed increased transcripts of OB differentiation markers by quantitative RT-PCR (n = 3/genotype). Data is represented as fold change following normalization to cyclophilin levels. RUNX2 = cbfa1, OSX = osterix, ALP = alkaline phosphatase, OCN = osteocalcin. Significance (*) indicates p<0.05 by Student's T-test. e) In vitro differentiation of OB from bone marrow stromal cells under osteogenic conditions (β-glycerophosphate and ascorbic acid). Cells were co-stained for alkaline phosphatase (ALP) expression (purple) and mineralization (Alizarin Red) at indicated days. Note the nearly complete mineralization (red stain) of *Arf^-/-^* wells present at each day as compared to punctate areas of mineralization present in *Arf* ^+/+^ wells. Representative of >3 independent experiments.

Bone remodeling is a coupled cycle of osteoblastic bone formation and osteoclastic bone resorption [11], [28], [29]. We previously demonstrated that *Arf^-/-^* mice have enhanced OC activity through ARF effects on nucleophosmin and ribosome biogenesis [8]. *p53* ^-/-^ mice have also been shown to have enhanced OC activity; however, this is attributed to an indirect (non-cell autonomous) increase in OB function resulting from p53 loss [9]. As the effect of ARF on OB function has not been characterized, we performed a series of experiments to further investigate its role in bone biology. MicroCT analysis of 8-week-old *Arf^-/-^* and *Arf* ^+/+^ littermates demonstrated significant increases in bone mineral density, trabecular bone volume and trabecular number and thickness in *Arf^-/-^* mice (**Figure 1a**). This increase in bone density was unexpected as the increased OC activity in *Arf^-/-^* mice would typically result in decreased bone density. To further investigate this paradox, we examined the OB compartment of *Arf^-/-^* mice. Bone formation rates, as measured by double calcein labeling, were significantly increased in *Arf^-/-^* mice (**Figure 1b**). Likewise, increased serum levels of osteocalcin, a biomarker of mature OB, were observed in *Arf^-/-^* mice (**Figure 1c**). Using quantitative real time-PCR, we found significantly increased expression of the OB differentiation markers osterix (OSX), alkaline phosphatase (ALP) and osteocalcin (OCN) (**Figure 1d**) in long bones of *Arf^-/-^* mice compared to littermate controls. As was observed in p53^-/-^ OB [9] no significant difference was seen in the expression of RUNX2 in the absence of ARF. Although RUNX2 has been reported to directly regulate *Arf*, with deficiency resulting in increased tumorigenic potential due to loss-of-senescence [30], the increased activity observed in *Arf^-/-^* OB was likely due to the absence of p53-mediated repression of osterix transcription [9]. Finally, OB differentiation was accelerated in *Arf^-/-^* mice in an *in vitro* differentiation assay. Primary OB generated from *Arf^-/-^* bone marrow stromal cells had increased ALP-positive cells and increased matrix mineralization, a function of mature OB, compared to cells from *Arf* ^+/+^ mice (**Figure 1e**), consistent with a direct cell autonomous effect of ARF loss in OB differentiation and activity. In conjunction with the previous report of enhanced OC activity in *Arf^-/-^* mice [8], these data demonstrate that *Arf^-/-^* mice have both increased osteoclastic bone resorption and osteoblastic bone formation which is consistent with increased bone remodeling.

### Loss of *Arf* led to development of osteosarcoma in Tax-transgenic mice

**Figure 2:**
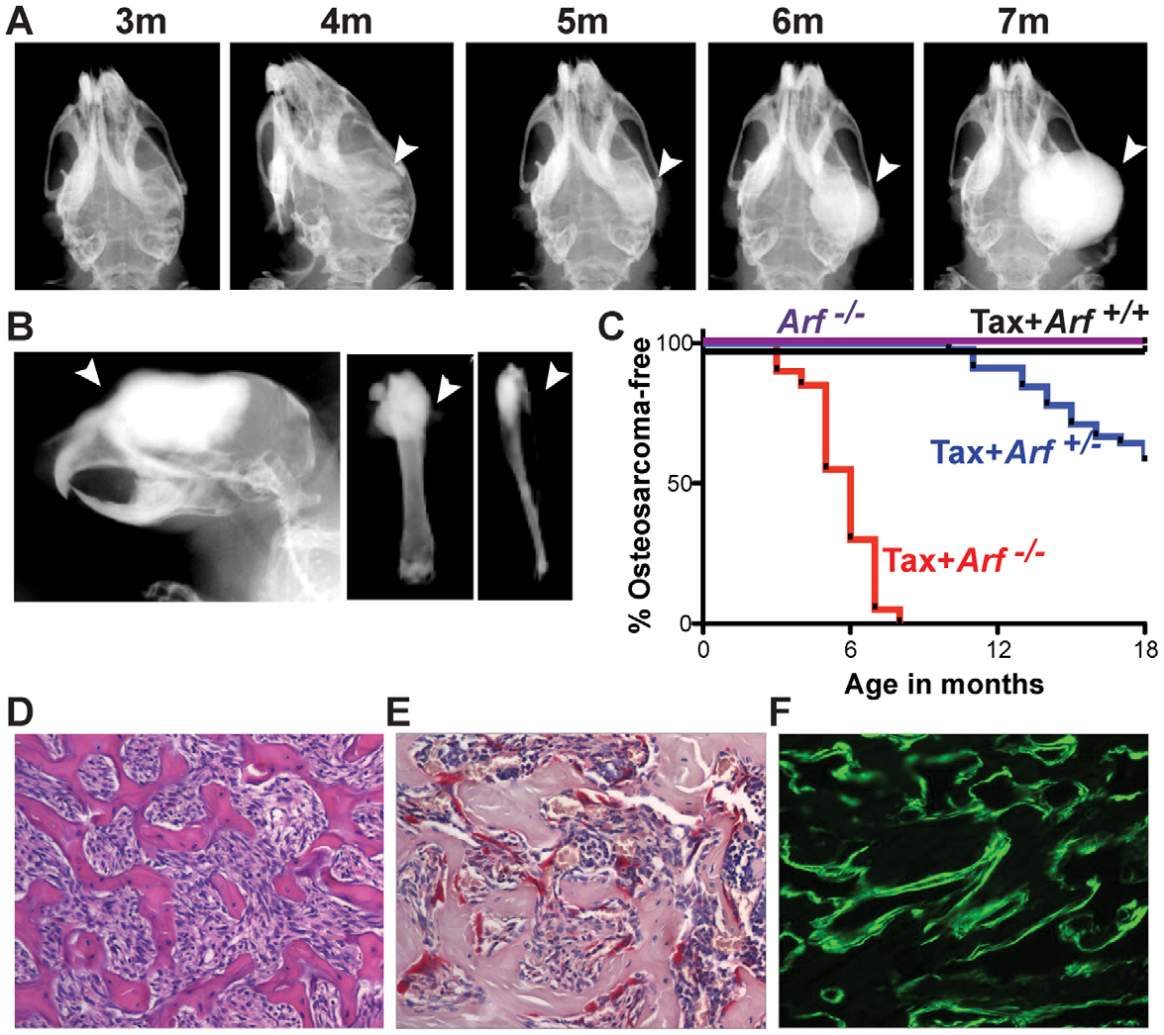
Loss of *Arf* led to development of high penetrance osteosarcoma in Tax-transgenic mice. a) Serial radiographs of a spontaneous OS on the right jaw of a representative Tax+*Arf^-/-^* mouse from 3 months to 7 months of age. b) Radiographs of OS on the skull (right), femur (middle) and tibia (right). c) Kaplan-Meier plot showing OS incidence in Tax+*Arf^-/-^* (red), Tax+*Arf* ^+/-^ (blue), Tax+*Arf* ^+/+^ (black) and *Arf^-/-^* (purple) mice. Note the decreased incidence and increased latency of OS in Tax+*Arf* ^+/-^ mice due to loss-of-heterozygosity. Tax+*Arf^-/-^* vs. Tax+*Arf* ^+/-^, Tax+*Arf* ^+/+^ and *Arf^-/-^*: p<0.001; Tax+*Arf^+/-^* vs. Tax+*Arf* ^+/+^ and *Arf^-/-^*: p = 0.002 d-f) Characterization of a representative Tax+*Arf^-/-^* mandibular OS. d) H&E. Note the trabeculae of bone formed by the OS (bright pink). e) TRAP staining demonstrating osteoclasts (red stained) lining OS bone surface. f) Double calcein labeling, 7d and 2d prior to sacrifice, showing sites of active mineralization.

We previously reported that mice transgenic for the HTLV-1 viral oncogene Tax spontaneously develop lymphocytic tumors associated with hypercalcemia, osteolytic bone lesions and enhanced OC activity [15], [16]. Because osteoclastic bone resorption and high bone turnover enhance tumor growth in bone [10], [11], we hypothesized that *Arf* loss in Tax mice would accelerate the growth and progression of tumors in the bone. We did not observe an acceleration in the development of lymphocytic tumors; however, nearly 100% of the Tax*+Arf* ^-/-^ mice unexpectedly developed OS (a tumor of primary OB) (**Figures 2a,b**). Calcified tumors were evident by radiography and/or palpation in Tax*+Arf^-/-^* mice by 7 months of age, while no OS were observed in Tax*+Arf* ^+/+^ or *Arf^-/-^* animals (**Figure 2c**). Notably, OS developed in Tax+*Arf^+/-^* mice with a penetrance of approximately 50% and a median onset of 14 months of age (**Figure 2c**). Tumors most frequently arose on the mandible, possibly due to the high rate of local bone remodeling at the root of the continuously erupting incisors [31]. Other mouse models of spontaneous OS demonstrate a predisposition to OS development in the mandible [18], [19]. We also observed OS in the maxilla and frontal bones of the skull in 50% of Tax+*Arf^-/-^* mice and in the femur or tibia in approximately 10% of mice (**Figure 2b** **, Figure S1**). Overall, the bone tumors consisted of well-demarcated, nodular, moderately cellular, unencapsulated masses that originated in bone and expanded into and replaced existing bone. The cells at the outer perimeter of the mass that were responsible for the expansile growth of the tumors were OB-like with a polygonal morphology and amphiphilic cytoplasm. The tumors were composed of trabecular bone interspersed with dense spindle cells that had eosinophilic and lightly vacuolated cytoplasm, ovoid nuclei, moderate anisocytosis/anisokaryosis and a low mitotic index (**Figure 2d**). We also observed substantial numbers of OC on the surface of the newly formed pathological bone (**Figure 2e**). Serial radiological imaging (**Figure 2a**) and double calcein labeling (**Figure 2f**) showed a rapid rate of new bone growth in the Tax+*Arf^-/-^* tumors. These data demonstrate that Tax+*Arf^-/-^* mice develop high penetrance spontaneous OS.

### Functional Tax protein and biallelic *Arf* loss were necessary for osteosarcoma development in Tax+*Arf^-/-^* mice

**Figure 3:**
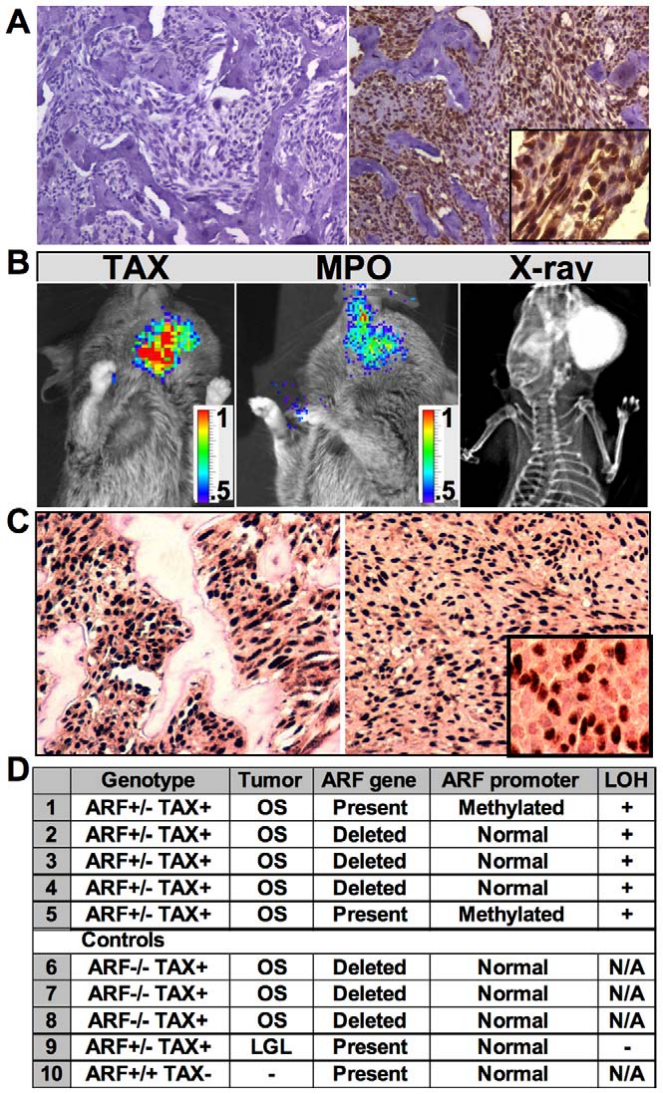
Functional Tax protein and biallelic *Arf* loss were necessary for osteosarcoma development in Tax+*Arf^-/-^* mice. a) Immunohistochemical staining of OS section for Tax (right) compared to isotype control (left). b) In vivo bioluminescence. (Left) HTLV-1 LTR-driven bioluminescent activity as a marker of Tax activity in OS. (Middle) Luminol-based detection of myeloperoxidase (MPO) activity. (Right) Radiograph. Color scale indicated is X10^4^ photons/s/cm^2^/sr. c) Immunohistochemistry for nucleophosmin (NPM) in primary Tax+*Arf^-/-^* OS. 2 representative samples are shown. d) Genetic evaluation of the *Arf* locus for loss-of-heterozygosity (LOH) in Tax+*Arf^+/-^* OS (top panel), control Tax+*Arf^-/-^* OS, Tax+*Arf^-/-^* large granular lymphocytic (LGL) peripheral tumor and Tax+*Arf^+/+^* tumor-free tail tissue.

The Tax oncoprotein in this model is regulated by the human granzyme B promoter which targets expression primarily to activated T cells and NK cells. However, Tax transcript was also detected in non-transformed OB cultured from Tax+ mice. Moreover, the Tax oncoprotein was detected in OS tumor cells using immunohistochemical staining (**Figure 3a**). To determine if the Tax protein expressed in the tumor retained its normal function and specificity as a transcriptional transactivator, we generated triple transgenic TAX-LUC-*Arf^-/-^* mice in which the HTLV-1 LTR drives expression of firefly luciferase in tissues in which Tax is expressed [22]. Using bioluminescent imaging to detect light emission in live mice, we found elevated LTR activity (a reporter for functional Tax activity) in the OS tumors (**Figure 3b**). Tax expression is known to recruit inflammatory cells and OC in lymphoid tumors [16], [22], [32]. Luminol-mediated bioluminescence was used to non-invasively detect myeloperoxidase (MPO) activity which is associated with tumor-infiltrating neutrophils and inflammatory cells [33]. Bioluminescence imaging using Luminol detected elevated MPO activity in the Tax+*Arf^-/-^* OS tumors consistent with immune cell infiltration in the OS tumors (**Figure 3b** **)**.

Genetic evaluation of the *Arf* locus in the tumors from Tax+*Arf* ^+/-^ mice demonstrated loss of heterozygosity (LOH) of the remaining *Arf* allele in all OS tumors tested from Tax+*Arf* ^+/-^ mice (n = 5), two by promoter methylation and three by genetic deletion (**Figure 3d**). DNA extracted from tumor-free tail tissue or from non-OS peripheral lymphocytic tumors retained the remaining *Arf* allele in the heterozygous animals indicating that LOH correlated with OS development, but did not correlate with development of large granular lymphocytic lymphoma in the same animal (**Figure 3d**). Finally, immunohistochemical staining demonstrated that expression of nucleophosmin (NPM), an oncogene regulated by ARF, was elevated within a sub-population of cells present in OS tumors (**Figure 3c**). From these studies, we concluded that loss of both *Arf* alleles was critical to OS development. Furthermore, functional Tax protein and NPM, a gene repressed by ARF, were expressed in the OS cells. Finally, there was robust cellular recruitment to these tumors, consisting of OC, inflammatory and immune cells.

### Orthotopic inoculation of a Tax+*Arf^-/-^* osteosarcoma cell line induced mineralized tumors

**Figure 4:**
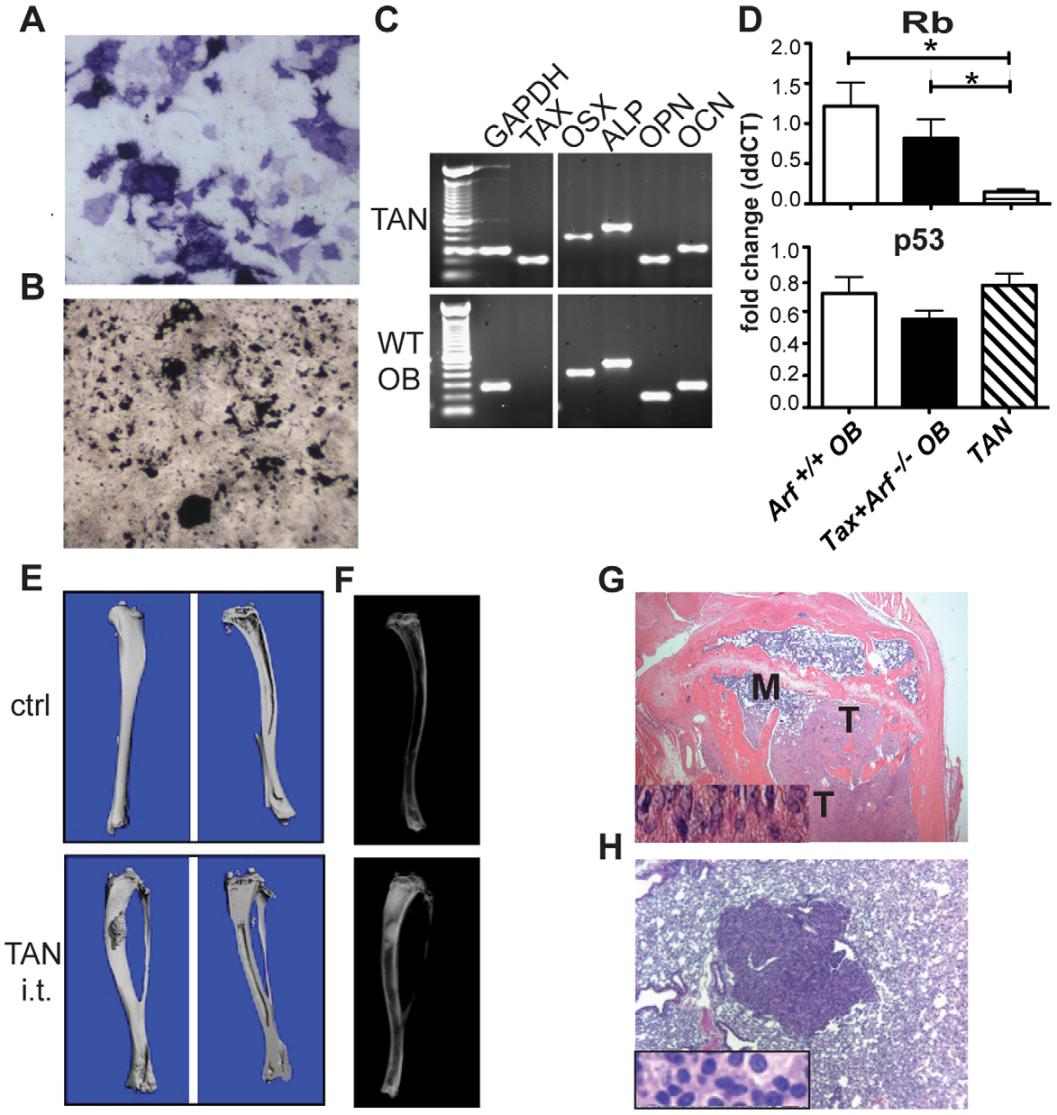
Orthotopic inoculation of a Tax+*Arf^-/-^* osteosarcoma cell line induced mineralized tumors. a) Alkaline phosphatase (ALP, purple) staining of Tax+*Arf*-null (TAN) OS line. b) von Kossa staining (black) showing mineralization of TAN cells after 10 days of culture under osteogenic conditions. c) TAN cells express mature OB markers by RT-PCR. OSX = osterix, ALP = alkaline phosphatase, OPN = osteopontin, OCN = osteocalcin. d) Quantitative RT-PCR for Rb (top) and p53 (bottom) expression in d20 in vitro differentiated OB from *Arf* ^+/+^ (open bars), Tax+*Arf^-/-^* (closed bars), or TAN OS cells (hatched bars). Data is represented as fold change following normalization to cyclophilin levels. Significance values of Rb: *Arf* ^+/+^ OB vs TAN p = 0.0109; Tax+*Arf^-/-^* OB vs TAN p = 0.0287. e–g) TAN cells form tumors when transplanted intratibially into immunocompromised NSG mice. e) 3D reconstruction from microCT analysis of contralateral control tibia (top) and TAN-injected tibia (bottom). Arrowheads note area of abnormal mineralization. f) Radiographs of tibias as in (e). g) H&E. M = marrow, T = tumor. Inset shows high power magnification demonstrating pathological osteoid deposition (light pink). h) TAN cells metastasize to lung following intravenous transplant. H&E staining of a metastatic lung lesion, inset shows high power magnification.

Similar to *Arf^-/-^* OB (**Figure 1e** **)**, Tax+*Arf^-/-^* OB showed increased differentiation and mineralization in vitro (**Figure S2**). The presence of Tax slightly enhanced OB differentiation when compared to wild-type cells and caused a modest increase in Tax+*Arf^-/-^* OB differentiation relative to *Arf* deficiency alone. To identify and characterize the malignant cell population within the OS we established a cell line, herein referred to as TAN (Tax *Arf* Null), from a primary mandibular OS of Tax*+Arf^-/-^* mice. TAN cells were alkaline phosphatase positive (**Figure. 4a**) and when cultured under osteogenic conditions were capable of producing mineralized matrix as measured by von Kossa staining (**Figure 4b**). In vitro, TAN cells expressed the OB differentiation markers osterix, alkaline phosphatase, osteopontin and osteocalcin, as well as Tax (**Figure 4c**). The TAN cells had intact levels of p53 mRNA and reduced Rb mRNA (**Figure 4d**) as was observed in the primary OS tumors **(Figure S3)**. After intratibial inoculation, TAN cells formed radiodense tumors that could be visualized by micro-CT (**Figure 4e**) and radiography (**Figure 4f**) in 5 out of 10 mice at 8 weeks following transplantation. Histological examination revealed abundant tumor cells interspersed within immature osteoid (**Figure 4g**). Additionally, intravenous inoculation of TAN cells resulted in multifocal lung metastases that exhibited histology consistent with a poorly differentiated sarcoma (**Figure 4h**). Collectively, these data demonstrate that TAN tumor cells were of OB origin with the ability to form mineralized tumors and metastasize upon transplantation, which are key characteristics of OS. It also suggests that RB may play a role in OS development in this model and shows that the deletion of *Arf* did not affect the mRNA expression of p53, consistent with other *Arf*-deficient mouse tumor models [5], [34].

#### Zoledronic acid administration prevented osteosarcoma development in Tax-Arf^-/-^ mice

**Figure 5:**
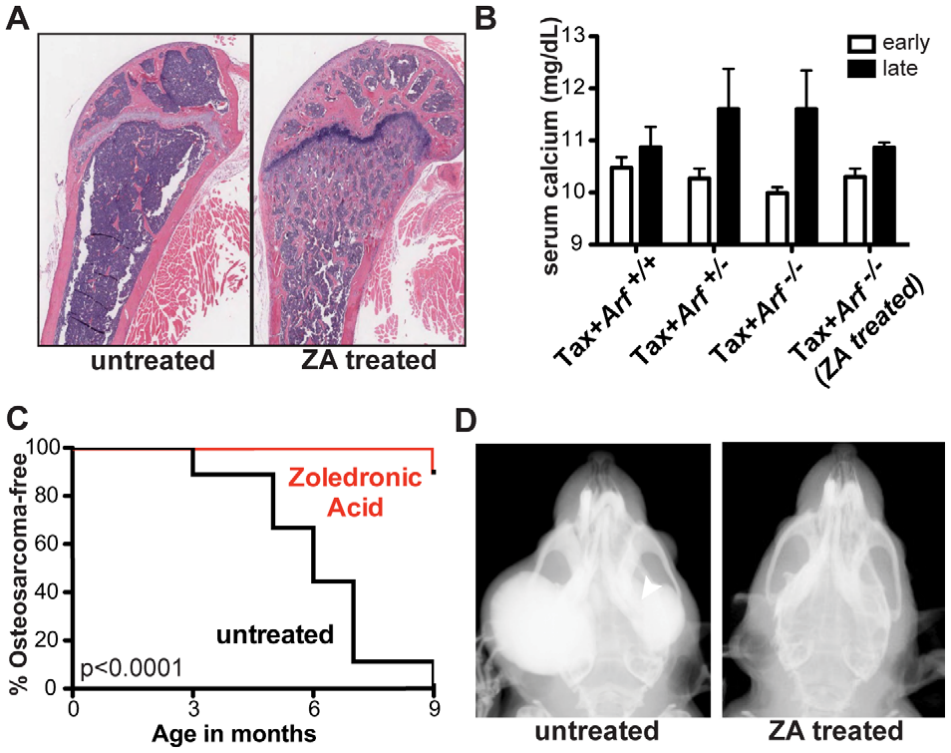
Zoledronic acid administration prevented osteosarcoma development in Tax+*Arf* *^-/-^* mice. Tax+*Arf^-/-^* animals were treated with 0.75 ug of zoledronic acid (ZA) once weekly from 1 month until 9 months of age. Appearance of OS was monitored monthly by manual examination and radiography. a) ZA treatment reduced OC bone resorption as shown by high trabecular bone volume. b) ZA treatment prevented hypercalcemia in Tax+*Arf^-/-^* mice. c) Incidence of OS. p<0.0001 by Log-rank test. d) Representative radiographs of 9-month-old Tax+*Arf^-/-^* mice untreated (left) or treated with ZA (right).

OS most commonly occurs in adolescent children during times of bone growth and high bone remodeling. We hypothesized that the increased bone remodeling in the Tax+*Arf^-/-^* mice was in part responsible for OS development in this model. Zoledronic acid (ZA), a drug commonly used for the treatment of osteoporosis and to prevent skeletal complications of bone metastasis, is a amino-bisphosphonate that blocks the activity of farnesyl pyrophosphate synthase (FPPS), a key component in the HMG-CoA reductase pathway, which is necessary for prenylation and lipid modification of a variety of proteins including Ras, Rho and Rac [35], [36]. Bisphosphonates bind to bone with high affinity, inhibit OC-mediated bone resorption and uncouple the cycle of bone remodeling [37], but do not significantly alter bone formation rates in mice [27]. We treated a cohort of Tax*+Arf^-/-^* mice with a weekly injection of ZA from one month of age to 9 months of age. This dosing regimen of ZA effectively reduced OC function and resulted in the development of high bone mass osteosclerosis **(** **Figure 5a** **).** In Tax mice, early and late stages of lymphoma can be distinguished by the complete blood counts and differential counts with elevated WBC and immature neutrophils present in the blood in late stage disease [14]. As expected, ZA protected the cohort from Tax-mediated hypercalcemia associated with late stage lymphoma (**Figure 5b**). Strikingly, ZA treatment prevented or delayed OS development in 8 of 9 mice (**Figures 5c and 5d**). These data indicate that early administration of bisphosphonates abrogated OS development in Tax+*Arf^-/-^* mice.

## Discussion

Bone remodeling is the continuous process of resorption and new bone formation that occurs in a healthy adult at the rate of about 10% per year [29]. The co-regulation of OC and OB is critical for balanced remodeling and healthy bone development. This current study implicates the ARF tumor suppressor as a critical regulator of normal bone remodeling. In the absence of ARF, the rate of bone remodeling is significantly accelerated and the activity of both OB and OC is increased. The dramatic increase in bone formation is reciprocally balanced by enhanced bone resorption, resulting in only modest osteosclerosis in *Arf^-/-^* mice. These findings support both p53-dependent and p53-independent actions of ARF on bone remodeling. OC activity is increased in *Arf^-/-^* mice in part through effects on NPM mediated ribosomal biogenesis [8] while OB activity was enhanced in the *Arf^-/-^* mice in a p53-dependent manner similar to that observed in the *p53^-/-^* mice [9].

The loss of ARF selectively predisposed Tax transgenic mice to development of OS. In our study, 100% of Tax+*Arf^-/-^* mice developed OS compared to 0% of animals with either modification alone. *Arf*-deficient mice rarely develop spontaneous OS [4]. Although the absence of *Arf* was sufficient to promote increased OB activation, proliferation and differentiation, other genetic hits are likely required to promote OB transformation. p14*^Arf^* is repressed by promoter methylation in 47% of human OS [20] and mutated or deleted in approximately 9% of human OS [17]. Moreover, *Arf* mutations and modifications are associated with decreased survival in patients with OS [20].

ARF is a well-established regulator of p53. Mice with OB specific loss of both p53 and Rb also develop spontaneous OS. Osterix-cre transgenic mice with p53 and Rb deletions in OB lineage cells have revealed that loss of *p53* is the rate-limiting step in initiation of OS, but that *Rb* loss cooperates in the steps leading to overt malignancy [18], [19]. The malignancy that arises in the Tax+*Arf^-/-^* model is comparable to that reported in these mouse models of OS. The loss of ARF compromises p53 function with OB no longer able to activate p53 in response to oncogenic stimuli [21], [38]. Previous studies have not observed OS development in Tax+*p53^-/-^* or Tax+*p53^+/-^* mice [39], however this is likely due to the severity of generalized tumor development causing mortality prior to the age when OS would develop.

MDM2, which is repressed by ARF, has been shown to negatively regulate Rb [40], [41]. While OS has been observed rarely in *Arf^-/-^* mice (approximately 2%) [4], we did not observe OS development in the *Arf^-/-^* mice on a Tax-null background (**Figure** 2c). This suggests that MDM2-mediated suppression of Rb was not likely sufficient to drive OB transformation in these mice. Interestingly, the viral oncogeneTax has been shown to repress Rb-like other viral oncogenes such as adenovirus E1A, SV40 large T antigen, HPV-16 E7 and/or HCMV pp71 by binding to RB protein and targeting it for degradation.

Although granzyme B, whose promoter drives Tax expression in this model, was originally described to be restricted to T and NK populations, recent data suggests that its expression may be induced in a wider variety of normal and malignant cells, including articular chondrocytes [42] which are derived from the same progenitor cells as OB. Thus, expression of Tax in malignant OB could be the result of a technical artifact such as promoter leakiness or could be revealing previously unrealized granzyme B promoter activity during OB differentiation. Tax is also a transcriptional activator that regulates cytokines and growth factors and recruits inflammatory cells and OC to Tax-expressing tumors [14], [16]. Indeed, we observed increased myeloperoxidase activity and prominent OC numbers in the OS tumors. This model can be used to better understand the role of tumor-associated stroma in OS, which could offer new therapeutic possibilities in a tumor that has been typically resistant to many chemotherapeutic agents.

Tax constitutively activates both the canonical and non-canonical arms of the NFκB pathway. Inhibition of the NFκB pathway has been shown to target OS cells lines [43] and reduce their metastatic potential [44], [45]. Interestingly, cisplatin has been recently described as an ARF mimic in OS therapy as it represses signaling through the NFκB pathway via the same mechanism as ARF [46]. Therefore it is conceivable that the loss of ARF and gain of Tax in Tax+*Arf^-/-^* mice has uncovered a role for the NFκB pathway in OS for which new therapeutics such as proteosome inhibitors could be investigated. The role of tumor associated stroma and inflammation is an emphasis in modern cancer biology and this model will allow further interrogation of the role these pathways in OS.

The connection between increased bone remodeling and OS has spurred research and ongoing clinical trials examining whether bisphosphonates are effective to treat OS. Although targeting OC may seem counterintuitive as a therapy for OS, within the context of living bone OC and OB regulate each other through “coupled” mechanisms. The therapeutic effect of zoledronic acid has been tested in allograft models with OS cell lines, treatment of mature tumors, and treatment of OS cells in culture (reviewed in [47], [48], [49]). Furthermore, zoledronic acid decreased Ewing primary bone tumor growth in mice [50], suggesting that such therapy may be effective against multiple types of primary bone cancer. To date, at least six clinical trials have been initiated to investigate the efficacy of bisphosphonates, as a single agent or coupled with conventional chemotherapies, in treating human OS. The therapeutic affect of OC inhibition through RANKL targeting has also been tested in established preclinical models of OS and Ewing's sarcoma [51], [52]. The effect of RANKL inhibition on prevention of OS development in the Tax+*Arf^-/-^* model is underway.

Currently, most research has focused on the treatment of established OS. However, in a model of spontaneous OS, our data reveal a role for bisphosphonates in the prevention or delay of OS development. Thus, prophylactic treatment with bisphosphonates, which have safely and effectively been used to treat Paget's disease of the bone [53], could benefit patients at elevated risk of developing OS or could be used as an adjuvant therapy after removal of a primary OS lesion. In addition to the increased risk of developing OS secondary to radiation therapy, several genetic cancer syndromes exist that place affected people at high risk for OS development. Alterations and mutations in the p53, retinoblastoma (RB) or neurofibromatosis1 (NF1) pathways predispose patients to OS [54], [55], [56] and components are these pathways are commonly mutated in spontaneous human OS [17]. Notably, the incidence of OS is markedly increased in patients with hereditary retinoblastoma [57] and in patients with the autosomal dominant Li Fraumini p53 mutations [54]. The diminished cost of genomic sequencing has enabled more testing for cancer predisposition syndromes. For example *NF1* mutations occur in 1 in 1000 people and NF1 patients are at increased risk for OS [58]. Our data would suggest that clinical trials to evaluate agents like zoledronic acid in the prevention of OS in high-risk populations and to prevent recurrence and metastasis after resection/treatment of primary OS tumors should be considered [57]. Preclinical studies demonstrate that ZA can decrease prostate cancer osteoblastic bone metastasis and tumor growth [59]. Bisphosphonate administration to patients with localized breast cancer has been associated with improved disease-free survival, decreased metastasis, lower occurrence of new breast cancers and decreased numbers of bone marrow micrometastases [60], [61]. Here we report a role for bisphosphonates in the prevention of OS development in a high risk model, which would be difficult to evaluate using xenograft models of established OS and osteoblastic metastases.

In summary, our data suggest a role for ARF as a regulator of normal bone remodeling and malignant bone tumor formation. The sensitivity of OS development to repression of bone resorption broadens the range of potential therapeutic prevention targets for patients at high risk for OS development.

## Supporting Information

Table S1 **Primers utilized for qualitative and quantitative PCR.**
(DOC)

Figure S1 **Tax+** ***Arf^-/-^*** **osteosarcoma tumors arise primarily in jaw and skull bones.** The locations of n = 50 OS tumors arising in Tax+*Arf^-/-^* mice. The majority of tumors arose in the mandible, often presenting with malocclusion prior to palpable tumor. Other bones of the skull include the frontal, parietal and premaxillary lobes. Maxillary bones contain the upper incisors and upper molars. Tumors occasionally arise in the long bones of the legs, however, these arise much later than the tumors of the skull and jaw.
(TIF)

Figure S2 **The expression of Tax did not cause a further significant increase in Tax+** ***Arf^-/-^*** **osteoblast differentiation relative to** ***Arf*** **deficiency alone.** In vitro differentiation of *Arf^+/+^*, Tax+, *Arf^-/-^* and Tax+*Arf^-/-^* OB from primary bone marrow stromal cells under osteogenic conditions (β-glycerophosphate and ascorbic acid). Cells were co-stained for alkaline phosphatase expression (purple) and mineralization (Alizarin Red) at indicated days. Representative of >3 independent experiments. Note that the experiment presented here is the same presented in **Fig 1e**.
(TIF)

Figure S3 **Tax+** ***Arf^-/-^*** **primary osteosarcoma have reduced levels of Rb transcript but intact levels of p53 transcript.** Quantitative RT-PCR for Rb (**A**) and p53 (**B**) expression in *Arf* ^+/+^ tumor-free bone (open bars), Tax+*Arf^-/-^* tumor-free bone (closed bars) or Tax+*Arf^-/-^* primary mandibular OS (hatched bars). N = 3/group. Data is represented as fold change following normalization to cyclophilin levels. Significance values of Rb: *Arf* ^+/+^ normal bone vs Tax+*Arf^-/-^* OS p = 0.0027; Tax+*Arf^-/-^* normal bone vs Tax+*Arf^-/-^* OS p = 0.0004.
(TIF)
