# Supplementary material for: The ARF Tumor Suppressor Regulates Bone Remodeling and Osteosarcoma Development in Mice
Source: PLoS One. 2010 Dec 30;5(12):e15755. doi: 10.1371/journal.pone.0015755 (PMC3012707; doi:10.1371/journal.pone.0015755)
Supplement: Table S1 — Primers utilized for qualitative and quantitative PCR. (DOC) [file pone.0015755.s001.doc]

**Table S1**

| **Qualitative RT-PCR Primer (Figure 4c)** | |
| --- | --- |
| GAPDH Fwd | ACTTTGTCAAGCTCATTTCC |
| GAPDH Rev | TGCAGCCGAACTTTATTGATG |
| Tax Fwd | TTCGGATACCCAGTCTACGTGTT |
| Tax Rev | GGAGTCGAGGGATAAGGAACTGTA |
| Osterix Fwd | CTGGGGAAAGGAGGCACAAAGAAG |
| Osterix Rev | GGGTTAAGGGGAGCAAAGTCAGAT |
| AlkPhos Fwd | ATTGCCCTGAAACTCCAAAACC |
| AlkPhos Rev | CCTCTGGTGGCATCTCGTTATC |
| Osteopontin Fwd | TCTGATGAGACCGTCACTGC |
| Osteopontin Rev | AGGTCCTCATCTGTGGCATC |
| Osteocalcin Fwd | CTCTGTCTCTCTGACCTCACAG |
| Osteocalcin Rev | CAGGTCCTAAATAGTGATACCG |
|  |  |
| **Quantitative RT-PCR Primers (Figures 1d 4d, S2)** | |
| Cyclophilin Fwd | AGCATACAGGTCCTGGCATC |
| Cyclophilin Rev | TTCACCTTCCCAAAGACCAC |
| RUNX2 Fwd | AGCAACAGCAACAACAGCAG |
| RUNX2 Rev | GCTCACGTCGCTCATCTTG |
| Osterix Fwd | CCCTTCTCAAGCACCAATGG |
| Osterix Rev | AAGGGTGGGTAGTCATTTGCATA |
| AlkPhos Fwd | ACACCAATGTAGCCAAGAATGTCA |
| AlkPhos Rev | GATTCGGGCAGCGGTTACT |
| Osteocalcin Fwd | CAGCGGCCCTGAGTCTGA |
| Osteocalcin Rev | GCCGGAGTCTGTTCACTACCTTA |
| Rb Fwd | TGCATCTTTATCGCAGCAGTT |
| Rb Rev | GTTCACACGTCCGTTCTAATTTG |
| p53 Fwd | GCGTAAACGCTTCGAGATGTT |
| p53 Rev | TTTTTATGGCGGGAAGTAGACTG |
