# Supplementary figures and images for: The ARF Tumor Suppressor Regulates Bone Remodeling and Osteosarcoma Development in Mice
Source: PLoS One. 2010 Dec 30;5(12):e15755. doi: 10.1371/journal.pone.0015755 (PMC3012707; doi:10.1371/journal.pone.0015755)

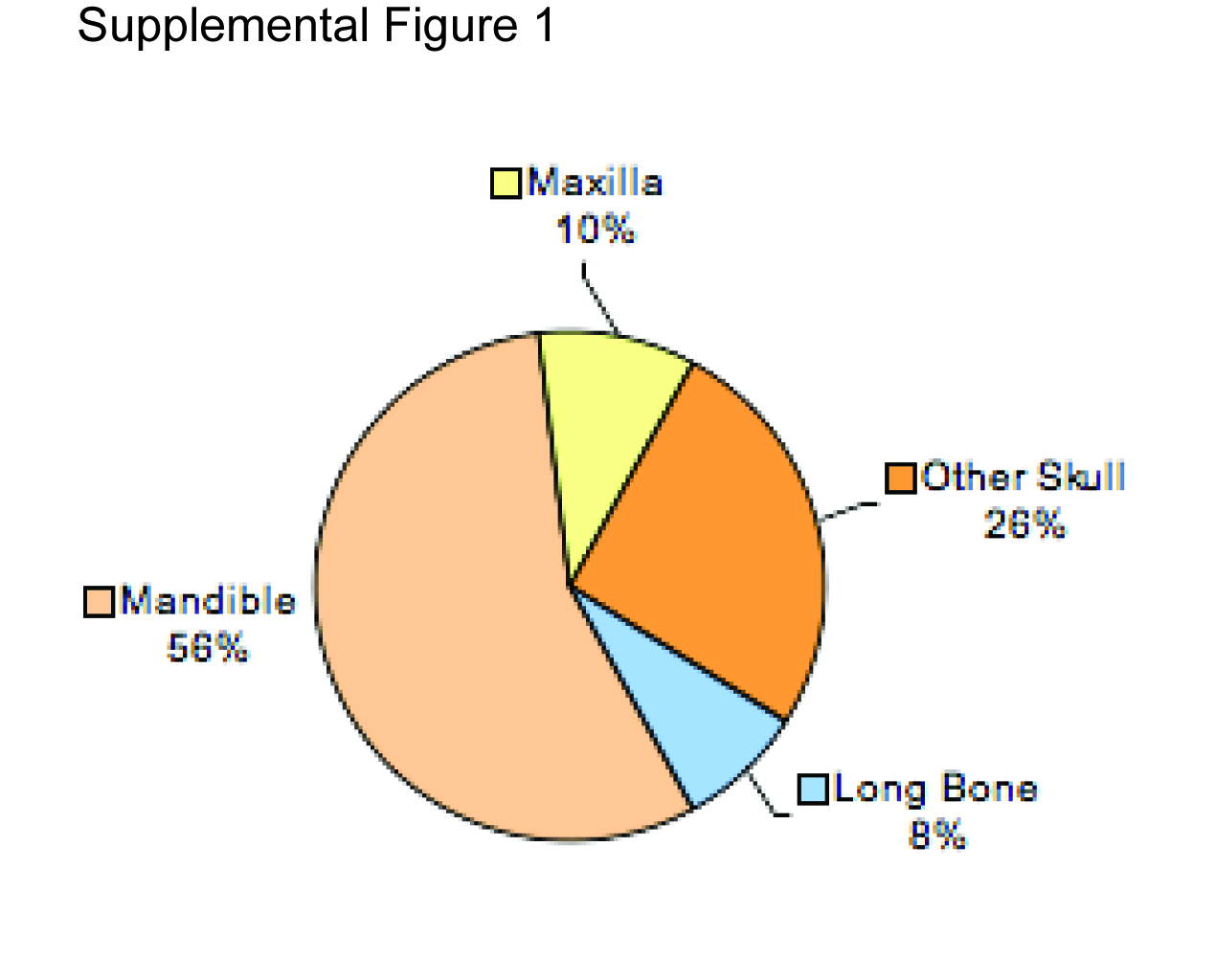

Supplement: Figure S1 — Tax+ Arf-/- osteosarcoma tumors arise primarily in jaw and skull bones. The locations of n = 50 OS tumors arising in Tax+Arf-/- mice. The majority of tumors arose in the mandible, often presenting with malocclusion prior to palpable tumor. Other bones of the skull include the frontal, parietal and premaxillary lobes. Maxillary bones contain the upper incisors and upper molars. Tumors occasionally arise in the long bones of the legs, however, these arise much later than the tumors of the skull and jaw. (TIF) [file pone.0015755.s002.tif]

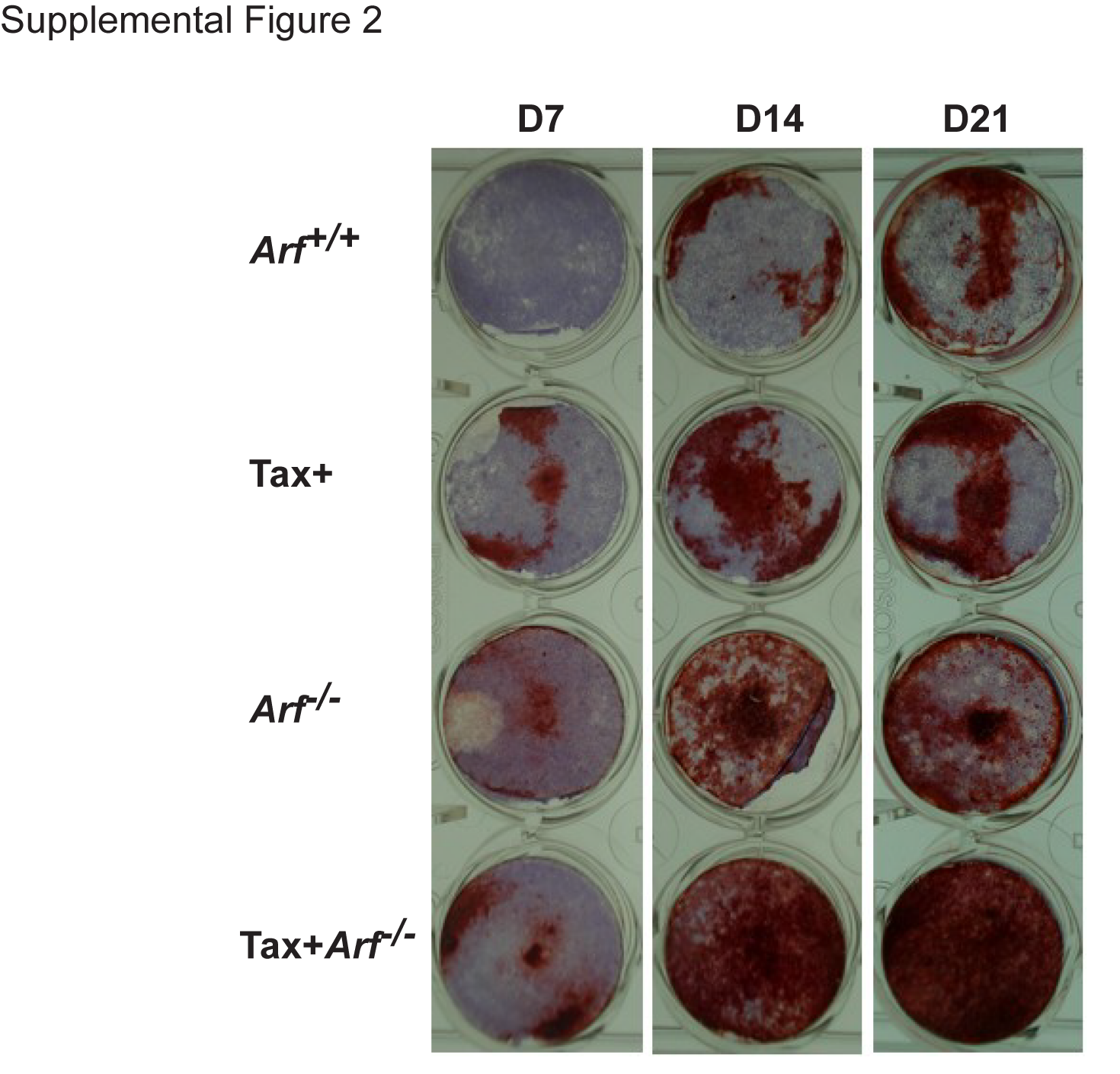

Supplement: Figure S2 — The expression of Tax did not cause a further significant increase in Tax+ Arf-/- osteoblast differentiation relative to Arf deficiency alone. In vitro differentiation of Arf+/+, Tax+, Arf-/- and Tax+Arf-/- OB from primary bone marrow stromal cells under osteogenic conditions (β-glycerophosphate and ascorbic acid). Cells were co-stained for alkaline phosphatase expression (purple) and mineralization (Alizarin Red) at indicated days. Representative of >3 independent experiments. Note that the experiment presented here is the same presented in Fig 1e . (TIF) [file pone.0015755.s003.tif]

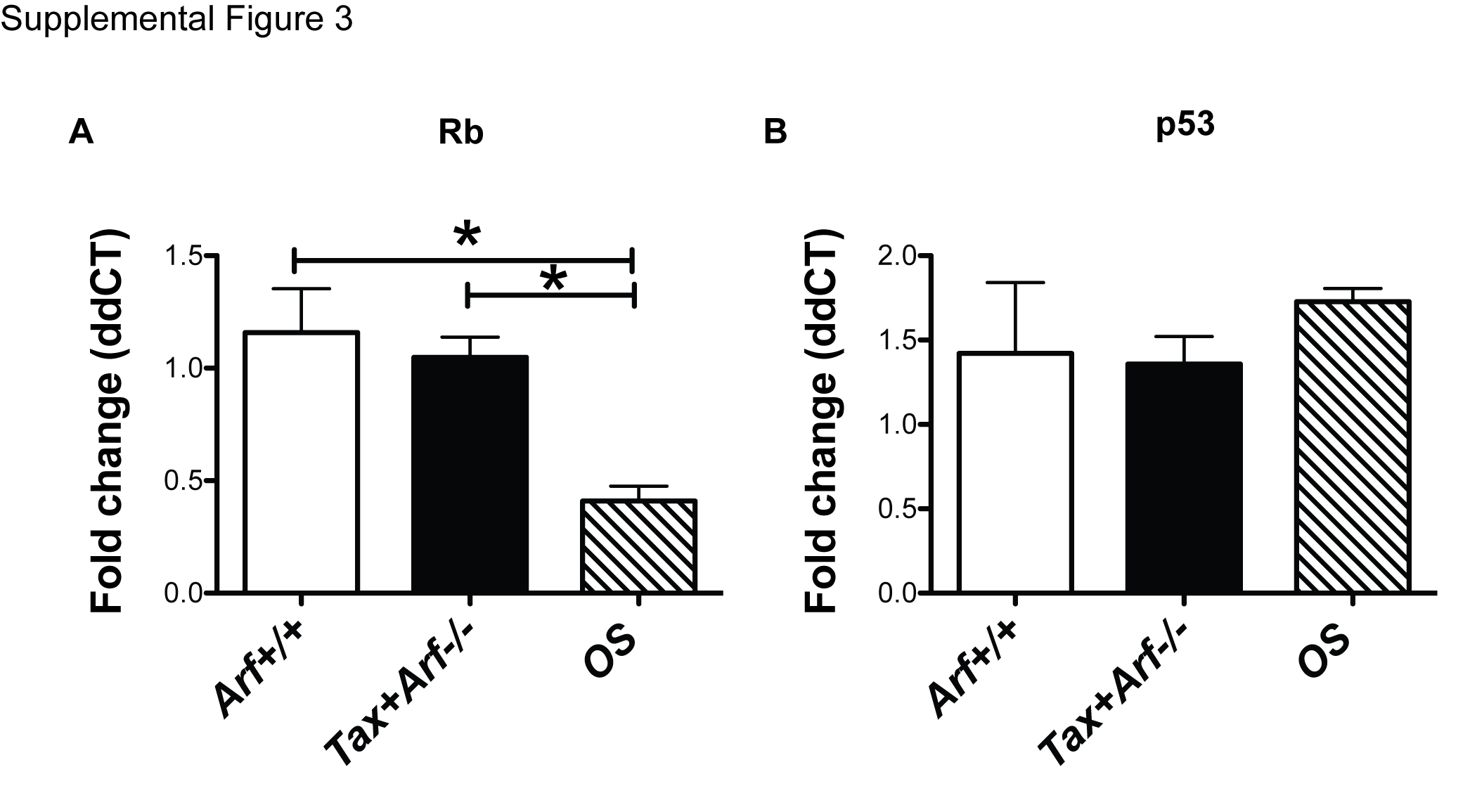

Supplement: Figure S3 — Tax+ Arf-/- primary osteosarcoma have reduced levels of Rb transcript but intact levels of p53 transcript. Quantitative RT-PCR for Rb (A) and p53 (B) expression in Arf +/+ tumor-free bone (open bars), Tax+Arf-/- tumor-free bone (closed bars) or Tax+Arf-/- primary mandibular OS (hatched bars). N = 3/group. Data is represented as fold change following normalization to cyclophilin levels. Significance values of Rb: Arf +/+ normal bone vs Tax+Arf-/- OS p = 0.0027; Tax+Arf-/- normal bone vs Tax+Arf-/- OS p = 0.0004. (TIF) [file pone.0015755.s004.tif]
